# Factors affecting the consumption of iodized salt by pregnant women in Karachi

**DOI:** 10.12669/pjms.38.3.4991

**Published:** 2022

**Authors:** Faryal Shaikh, Syed Imtiaz Ahmed Jafry, Asad Ali Khan

**Affiliations:** 1 Dr. Faryal Shaikh, MBBS, MPH. Senior Lecturer in Department of Community Medicine, Fazaia Ruth Pfau Medical College, Karachi, Pakistan; 2 Prof. Dr. Syed Imtiaz Ahmed Jafry, MBBS, MPH. Head of Department of Community Medicine, Baqai Medical University, Karachi, Pakistan; 3 Prof. Dr. Asad Ali Khan, MBBS, FCPS. Head of Department in Community Medicine, Fazaia Ruth Pfau Medical College, Karachi, Pakistan

**Keywords:** Iodine Deficiency Disorder (IDD), Iodized Salt, Universal Salt Iodization (USI)

## Abstract

**Background and Objective:**

Globally iodine deficiency disorder (IDD) is a major preventable cause of cognitive impairment in new born. In developing countries, every year 38 million newborn develop cognitive impairment as a result of iodine deficiency. Iodine consumption by pregnant women is affected by many factors. Hence, we conducted this study to identify factors associated with IDD. To know the effects of different factors on use of iodized salt by pregnant women visiting antenatal outpatient department (OPD) at a public sector tertiary care hospital of Karachi.

**Methods:**

Pregnant women (n=360) visiting antenatal OPD at public sector tertiary care hospital of Karachi were interviewed using a structured questionnaire. Systematic random sampling method was employed. Data was collected from March 2017 to January 2018. Chi-square test was applied to identify factors associated with IDD.

**Results:**

Thirty one (31% ) of pregnant women were consuming iodized salt in their homes. The percentage of participants who heard about iodized salt for the first time was 24%. Twelve percent (12%) reported that price of iodized salt is more than that of normal salt. Ninety eight (98%) of pregnant women replied that they were not informed about the importance of iodine or iodine requirement during pregnancy by their doctor or health care provider. A statistically significant association was observed between educational status (p=0.001) and household income (p<0.001) with the use of iodized salt.

**Conclusion:**

Low education, low income of study participants are identified as factors related to limited consumption and incorrect practices related to the use of iodized salt. In order to address iodine deficiency, there is a need to not only sensitize the expectant mothers about the adverse outcome of maternal iodine deficiency for their unborn child but also to introduce an awareness program at the antenatal clinics by the Health Professional for the antenatal care seeking women. There is also an immense need of support from Government side as well to make strategies and policy changes at the national level to ensure the availability, accessibility and affordability of iodized salt.

## INTRODUCTION

 Iodine is essential for normal growth and fetal brain development. Iodine deficiency disorder (IDD) is a major public health concern globally and cognitive impairment of newborn caused by IDD is a preventable cause of iodine deficiency worldwide.^1^ Since iodine is required for the synthesis of thyroid hormone and inadequate intake of iodine results in insufficient synthesis of thyroid hormone which causes functional and developmental abnormalities. The consequences of IDD include mental retardation, goiter, growth retardation, reproductive failure, and increased childhood mortality. The most alarming consequence of IDD is on developing brain of the infant.^2^ Therefore, in order to prevent and control IDD, iodized salt consumption is the most efficient and potent strategy.^3^

 Globally 1.88 billion people (28.5%) of world’s population have insufficient iodine intake and affected by iodine deficiency based on urinary iodine concentration and 37.4% of general population in Eastern Mediterranean region has inadequate iodine intake which includes Pakistan.^4^

 According to WHO, the daily intake of iodine recommended for pregnant women is 250µg in order to meet the requirements of iodine in pregnant and lactating women.^5^ In Pakistan, 47.7% of reproductive age women take less than 250µg and suffer from iodine deficiency.^6^ The reported use of iodized salt among women of reproductive age group (15-49 years) was around 40% which is alarmingly higher in urban areas (46.5%) than in rural areas (32.5%).^7^ Every year 38 million newborn suffer from the consequence of cognitive impairment as a result of iodine deficiency in developing countries including Pakistan.^8^ According to National Nutrition Survey of Pakistan (NNS), approximately 17.5% women of reproductive age (15-49years) and 15.7% of children aged (6-12 years) have low urinary iodine excretion. Among them, 7.3% of children had severe iodine deficiency while 23% of children were found to have moderate iodine deficiency.^9^

 Pregnancy influences greatly on thyroid function and thyroid gland. The physiological changes which take place during pregnancy are the 50% increase in production of thyroid hormone i.e. thyroxine (T4) and triiodothyronine (T3). This coupled with increased daily urinary excretion of iodine puts women in iodine deficient state, hence increasing daily requirement of iodine by 50%.^10^

 Therefore, this study was aimed to assess the effects of different factors on use of iodized salt by pregnant women visiting antenatal Outpatient department (OPD) at public sector tertiary care hospital of Karachi.

## METHODS

 A descriptive cross-sectional study was conducted in gynecology and obstetrics outpatient department (OPD) at Civil Hospital Karachi, Pakistan. A written permission was taken from the medical superintendent of the hospital. The study population included pregnant women aged 16-45 years seeking routine antenatal care and who were willing to participate in the study. Those who had endocrinological disorder, taking thyroid preparations, antidepressants (which may alter iodine levels in blood e.g. *Neurolith*) and those who did not give consent to participate in the study were excluded. A Systematic random sampling method was used in which every 5^th^ pregnant woman who were enrolled in the OPD register was recruited and then interviewed. Informed consent was taken from the study participants prior to an interview in which all the study participants were informed about the interview. A pretested questionnaire was administered to 360 pregnant women attending the antenatal care. After pretesting, relevant changes were made and the questionnaire was finalized. Sample size estimation was based on proportion of inadequate iodine intake (37.4%), taken from WHO-EMRO, with 5% level of significance and 95% confidence interval (CI). Using the above mentioned values, the calculated sample was found to be 360. Data was collected from March 2017 to January 2018. Data collection tool included a pretested structured questionnaire administered through face to face interview. Principal investigator interviewed the participants. There were two main sections in the questionnaire that included socio-demographic characteristics of pregnant women and factors associated with consumption of iodized salt among pregnant women. Study participants were asked about their age, religion, parity, ethnicity, family System, educational status, trimester, occupational status, monthly household income and socioeconomic status. Questions were also asked regarding the, sources, storage, consumption and practice of using iodized salt i.e. sprinkling during or after the cooking.

 Data was entered and analyzed using Statistical Package for Social Sciences (SPSS) version 22. Descriptive statistics were used to describe socio-demographic characteristics. Categorical variables were measured as percentages while age was expressed as mean and ± standard deviation.

(Household income and education)

1. Those who have an income of Rupees less than 7,000 (USD <45.51) or between Rupees 7000-17000.(USD=45.51-110.53) or are illiterate or have informal education are considered in low socioeconomic status.
2. Those who have an income of Rupees 18,000-30,000 (USD=117.03-195.05) or have primary or secondary level of education are categorized in Middle Socioeconomic status.
3. Those who have an income of greater than Rupees 30,000 (USD > 195.05) or have higher level of education are considered in Upper Socioeconomic Status.

1 USD=153.80 PKR on Monday, May 17, 2021

### Ethical Consideration

The plan of survey including (survey design, sampling technique) was reviewed and approved by the Ethical Review Committee of Baqai Medical University, Karachi, Pakistan. The personal information of all the participants was kept confidential. The informed consent was secured from each study respondent at the beginning of interview. Confidentiality of data was ensured and data was secured by password.

## RESULTS

 The total number of pregnant women enrolled in the study were 360. Data for four cases could not be collected. The study participants were categorized into three age groups i.e. 16-25 years (50% (n=180/360), 26-35 years (40% (n=148/360) and 36-45 years (9% (n=32/360). The proportion of illiterate subjects was 45% (n=166/360) while 24% (n=86/360) had primary education, 17% (n=61/360) had secondary education and the remaining 7% (n=25/360) had acquired higher education. The major ethnic group was Muhajirs (31%). More than half of the study participants, 55% (n=198/356) had a household income between rupees 7000 -17,000. 95% (n=346/360) of pregnant women were housewives (Table-I).

**Table-I:** Socio-demographic Characteristics of Pregnant Women (n=360), Karachi, Pakistan.

Characteristic	Frequency (N)	Percentage (%)
***Age (years)***		
16-25	180	50
26-35	148	40
36-45	32	9
Mean Age	27 [± 5.966 ]	
***Religion***		
Islam	355	99
Christian	3	0.8
Hindu	2	0.6
***Para***		
1-3	291	81
4-7	61	17
8-11	8	1
***Gravida***		
1-3	217	59
4-6	114	32
7-9	29	7
***Ethnic group***		
Punjabi	17	5
Saraiki	6	2
Muhajirs	116	31
Sindhi	52	13
Balochi	40	10
Pakhtoon	55	14
Others	77	21
***Family system***		
Nuclear	58	15
Joint	302	84
***Educational status***		
Illiterate	166	45
Informal Education	22	5
Primary	86	24
Secondary	61	17
Higher education	25	7
***Trimester***		
First	94	25
Second	110	31
Third	156	42
***Occupational status***		
House wife	346	95
Self employed	13	4
Employed	1	0.3
***Employment status of husband***		
Employed	338	94
Self employed	11	2
Unemployed	11	2
Monthly household income *		
Less than 7000	99	27.5
7000-17000	198	55.0
18000-30000	52	13
More than 30,000	7	2
***Socio economic status***		
Upper	2	0.6
Middle	44	11
Low	314	86

* n=356.

 About 68% (n=244/360) of the respondents did not consume iodized salt. Of these 24% (n=86/244) heard about iodized salt for the first time. The price of iodized salt is more than that of regular salt was reported by 12% (n=48/244) of the respondents. Around 98% of pregnant women replied that they were not informed about the importance of iodine or iodine requirement during pregnancy by their doctor or provider. (Table-II).

**Table II:** Consumption of iodized salt among pregnant women (n=360), Karachi, Pakistan.

Items in questionnaire	Frequency (N)	Percentage (%)
***Do you use iodized salt in your home?***		
Yes	116	31
No	244	68
***Why you do not use iodized salt?***		
The price of iodized salt is more than that of normal salt	48	12
The iodized salt has negative impact on reproduction (infertility)	8	1
The family member does not advice it.	33	8
Iodized salt differs in taste than normal salt	24	7
Iodized salt is not available easily	13	4
Heard about iodized salt first time	86	24
Iodized salt granules are thickened and does not dissolve easily	6	2
Others	15	3
Any or whatever available salt in the market	11	2
***Do you add iodized salt to your food since it prevents from iodine deficiency disorder?***		
Yes	16	3
No	97	27
***Does your health care provider or doctor inform you about importance of iodine or about iodine requirement during pregnancy?***		
Yes	8	2.2
No	352	98
***Do you use iodized salt during cooking or sprinkle it on top of the food?***		
During cooking	114	32
Sprinkle it after cooking	2	0.6
***Do you know that iodized salt is affected by sunlight, heat and moisture?***		
Sunlight	5	1.4
Heat	16	3
Moisture	28	8
All	9	2.5
No effect	12	2
Do not know	46	13
***Where do you store iodized salt in the kitchen?***		
Plastic container	28	8
Covered glass container	5	1.4
Air tight container (plastic) with fitting lid	82	23
Other	1	0.3
***Do you read labeling on salt bag when you buy salt to make sure that salt it is iodized?***		
Yes	60	17
No	56	16

 Pregnant women with higher household income and education were more likely to use iodized salt. There is no difference in consumption of iodized salt on the basis of ethnicity (Table-III).

**Table-III:** Association between Various Factors and Use of Iodized Salt in Pregnant Women, Karachi, Pakistan (n=360).

Characteristics	Use of Iodized Salt		X²	df	Critical value	p- value

	Yes (%)	No (%)				
Household income			19.776	2	5.991	< 0.001
Less than Rs.7,000	20(20.2)	79(80)				
Rs.7000-17000	62(31.3)	136(68.7)				
Rs.18000-30,000 or more	32(54.2)	27(46)				
Educational status			17.598	4	9.488	0.001
Illiterate	36(21.7)	130(78.3)				
Informal education	8(36.4)	14(63.6)				
Primary	33(38.4)	53(61.6)				
Secondary	26(42.6)	35(57.4)				
Higher Education	13(52)	12(48)				
Ethnic group			7.681	6	12.592	0.262
Punjabi	8(47.1)	9(52.9)				
Sariki	2(33.3)	4(66.7)				
Muhajirs	43(37.1)	73(62.9)				
Sindhi	12(23.1)	40(76.9)				
Balochi	10(25)	30(75)				
Pakhtoon	14(25.5)	41(74.5)				
Others	27(36.5)	47(63.5)				

## DISCUSSION

 The National IDD Control Program in Pakistan commenced in 1984 but it had little effect on utilization and availability of iodized salt.^11^ In 1993, Universal Salt Iodization (USI) has been recommended as a global strategy by WHO and UNICEF for the prevention of IDD which made considerable progress and implemented in more than 120 countries across the globe^12^. In 2006, Iodine deficiency disorder/Universal Salt Iodization Program (IDD/USI) in Pakistan was strengthened with the support of Micronutrient Initiative (MI) by the Ministry of Health (Nutrition Wing). In March 22, 2011, in order to improve and monitor salt iodization all across the country, Geographical Information System (GIS) was launched by Pakistan’s Ministry of Health.^11^ This added to the support given by government of Pakistan for IDD.

 Our results suggest that consumption of iodized salt has statistically significant association with household income and women’s education. Those women with primary, secondary and higher education level had higher chance of using iodized salt compared to uneducated one. Similarly, women with high household income level had higher chance of using iodized salt compared to women who had low education and low household income level. These findings are consistent with the different studies conducted.^13^-^17^ This increase consumption of iodized salt among educated and moderately high income women might be due to increased knowledge and awareness about the importance of using iodized salt among them. A study conducted in South Africa shows similar results that participants who belonged to low socioeconomic status were less informed about iodine nutrition as compared to participants from high socioeconomic status who were more likely to use iodized salt^13^. A study conducted in Mongolia reported a high level of consumption of iodized salt by more than 83% of respondents.^14^ This difference could be due to appropriate provision of information about importance and benefits of iodized salt from mass media, print media and medical personnel.

 Our study results are similar to a study conducted in Lahore, Pakistan of consuming iodized salt by pregnant women.^15^ In the present study low level of consumption of iodized salt is because of lack of awareness ,proper guidance, and absence of dissemination of information about iodine, IDD and iodized salt by the medical personnel, high price of iodized salt as compared to regular salt, lack of recommendation of iodized salt to overcome iodine deficiency by health professional (doctor, medical representative) while in a Mongolian study, mass media educational campaign and broadcasting of TV and radio program on iodized salt, IDD twice a week runs with the collaboration of Mongolian government and UNICEF office attributed for widespread consumption of iodized salt among pregnant women.^14^

 In a Sri Lankan study no association was seen between the practice of using iodized salt and the level of education. In the present study majority of pregnant women were unaware about the iodized salt and heard about it for the first time. They were not informed by the doctor or medical personnel regarding the consumption of iodized salt. Pregnant women were ignorant about the importance of iodized salt in diet for the prevention of iodine deficiency disorder. However, in a study conducted in Sri Lanka more than half of pregnant women were aware about the importance of iodine and received this information from the Public Health midwives and at the clinics during their antenatal visit.^16^

 Hence it is seen in a present study that the consumption of iodized salt was significantly higher in high education group and high income group. Hence the present study and a study conducted in Ethiopia, Tanzania and Bangladesh revealed similar results of association among the education level and income of participants with the use of iodized salt.^17^-^19^ According to a study conducted in Ethiopia reported that respondents who has a monthly income of 60 USD (1201 Ethiopia Birr) were about four times more likely to consume iodized salt as compared to those who have a low income and those who have formal education were about two times more likely to consume iodized salt as compared to those who had no formal education. In terms of taste and price of iodized salt, the present study and the Ethiopian study both showed similar results.^17^

 Our study findings are similar to the studies conducted in Tanzania and Bangladesh.^18^,^19^ This could be because of comparatively higher income and education levels in those countries.It is evident from this study that less education and low income of study participants are identified as factors related to limited consumption of iodized salt.

 In order to address iodine deficiency, there is a need for strong advocacy on the importance of iodized salt consumption to the pregnant women by the Health Professional and by making the pregnant women aware during the antenatal care about the increased requirement of iodine during pregnancy which is required for fetal brain development so that they can incorporate iodized salt in their diet, preventing themselves and their newborn child from the worst outcome and consequences of IDD.

### Limitations of Study

As this study was carried out in a single tertiary care hospital, therefore the results cannot be generalized at population level. Sample cards were shown to women to identify iodized salt correctly from the number of salts preparation available in market. This may have resulted in incorrect identification by some women.

## CONCLUSION

 The findings of this study would be useful to government at local, and federal level, which will help in organizing promotional and educational programs on the utilization of iodized salt by pregnant women. Provision of health education sessions by health care providers at the antenatal clinics should be ensured. There is also an immense need of support from Government side as well to make strategies and policy changes at the national level to ensure the availability, accessibility and affordability of iodized salt.

### Author’s Contribution:

**FS:** Conceived, designed, collected, entered, analyzed, interpret data and prepared the manuscript.

**IJ:** Reviewed, improvised the questionnaire, assisted in data analysis and interpretation.

**AK:** Prepared and critically review the manuscript and provided logical input for data analysis.

All authors reviewed and finalized approval of the manuscript.
